# Association Between Systemic Immune‐Inflammation Index and Sepsis‐Induced Liver Injury in Adult Patients With Sepsis: A Retrospective Cohort Study

**DOI:** 10.1002/iid3.70478

**Published:** 2026-06-30

**Authors:** Zimeng Qin, Jiaqi Li, Yijiang Liu, Beiyuan Zhang, Wenkui Yu

**Affiliations:** ^1^ Nanjing Drum Tower Hospital Clinical College of Nanjing Medical University Nanjing China; ^2^ Department of Critical Care Medicine, Affiliated Drum Tower Hospital Medical School of Nanjing University Nanjing China

**Keywords:** inflammatory biomarker, risk factors, sepsis, sepsis‐induced liver injury, systemic immune‐inflammation index

## Abstract

**Background:**

Sepsis‐induced liver injury (SILI) is a fatal complication of organ failure that currently lacks reliable biomarkers for early diagnosis and risk prediction. This study aimed to explore the link between the systemic immune‐inflammatory index (SII) and SILI in adult patients with sepsis in the intensive care unit (ICU) at Nanjing Drum Tower Hospital.

**Methods:**

This single‐center retrospective study analyzed the baseline characteristics of patients with sepsis admitted to the ICU between 2020 and 2024. Multifactorial logistic regression was used to identify independent risk factors for SILI. Curve fitting and Subgroup analyzes were performed to provide evidence for the stratified management of patients with different clinical characteristics. Receiver operating characteristic (ROC) curve analysis was performed to determine the predictive capacity of the SII.

**Results:**

A total of 231 patients were included, of whom 53 (22.9%) developed SILI during ICU hospitalization. Patients with SILI exhibited a significantly higher in‐hospital mortality rate than non‐SILI cases (52.8% vs. 25.3%, *p* < 0.001). Elevated SII at ICU admission was strongly associated with SILI development (*p* < 0.001). Multivariable logistic regression identified the SII as an independent risk factor for SILI. ROC curve analysis demonstrated that SII had superior predictive performance for SILI occurrence, with an area under the curve of 0.85 (95% CI: 0.784–0.907).

**Conclusion:**

This retrospective study revealed a substantial association between high SII and SILI in adult ICU patients with sepsis, suggesting that SII is a promising indicator for post‐sepsis SILI risk prediction.

## 1 Introduction

Sepsis is a remarkably complex and life‐threatening syndrome characterized by significant clinical heterogeneity, making diagnosis and treatment challenging [1, 2]. Sepsis is frequently associated with organ dysfunction, and hepatic function has emerged as a critical determinant of patient prognosis [3, 4]. Sepsis‐induced liver injury (SILI), in particular, is associated with substantial mortality as ICU‐managed cases reported in‐hospital mortality rate as high as 33% [4]. Therefore, early detection of SILI is imperative for the appropriate management of patients with sepsis. The pathogenesis of SILI involves a complex interplay between multiple pathophysiological pathways, and the underlying mechanisms remain under active investigation and require further elucidation. However, current research suggests that immune dysregulation and inflammatory responses are key contributors to the occurrence and progression of liver damage [5, 6, 7]. During sepsis, circulating pathogens and their metabolic products reach the liver via the bloodstream, activating toll‐like receptors on host immune cells and hepatic sinusoidal endothelial cells. This triggers an excessive inflammatory response, leading to hepatocellular injury and impaired metabolic and bactericidal liver functions [5]. Moreover, an imbalance in the immune response occurs, with activated immune cells releasing a plethora of pro‐inflammatory cytokines, including tumor necrosis factor‐alpha, interleukins, prostaglandins, and leukotrienes, leading to tissue inflammation and hepatic damage [6]. Concurrently, hepatocyte injury and death further amplify local and systemic immune‐inflammatory responses, exacerbating multi‐organ dysfunction [7]. Despite these insights, clinically accessible biomarkers for evaluating systemic inflammatory responses remain limited.

The systemic immune‐inflammation index (SII) has recently emerged as a reliable metric for evaluating systemic inflammatory responses and prognostic outcomes [8]. SII is calculated as (platelet count × neutrophil count)/lymphocyte count. Although initially introduced as a prognostic indicator for patients undergoing surgical intervention for hepatocellular carcinoma, SII has gained clinical utility due to its simple calculation, using standard hematological parameters, without requiring specialized assays. Over time, the SII has been increasingly adopted for the clinical evaluation of a vast array of pathological conditions. For example, in patients with ovarian cancer, higher preoperative SII values were linked to an increased risk of developing postoperative complications [9], making SII a proven parameter in identifying optimal therapeutic strategies for advanced ovarian cancer. Similarly, in a cohort of patients diagnosed with juvenile idiopathic arthritis between 2014 and 2023 [10], SII provided a more precise assessment of inflammation and disease activity, thereby demonstrating its clinical usefulness.

To date, no studies have systematically investigated the correlation between the SII and SILI. This study aimed to determine whether SII can serve as a predictive biomarker for SILI in critically ill adults with sepsis requiring ICU care.

## 2 Methods

### 2.1 Study Design and Population

**Figure 1:**
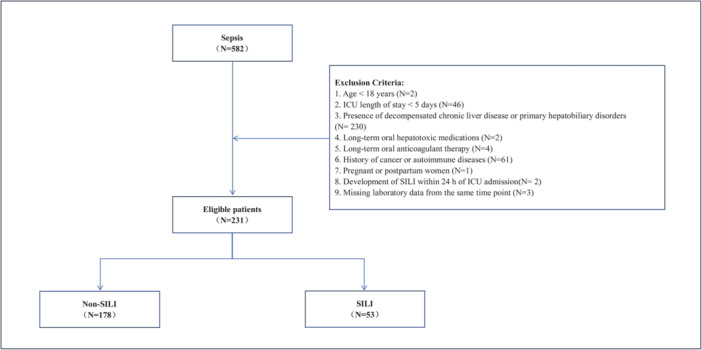
Flow chart of the study population.

This single‐center retrospective study included adult patients diagnosed with sepsis and admitted to the ICU at Nanjing Drum Tower Hospital, a tertiary academic medical center affiliated with Nanjing University, between January 2020 and January 2024. Initially, the study identified 582 patients meeting the Sepsis‐3 diagnostic criteria. The exclusion criteria were as follows: (1) age < 18 years; (2) ICU stay < 5 days; (3) decompensated chronic liver disease or primary hepatobiliary disorders; (4) long‐term use of oral hepatotoxic medications; (5) long‐term use of oral anticoagulant therapy; (6) history of cancer or autoimmune diseases; (7) pregnant or postpartum women; (8) SILI onset within 24 h of ICU admission; and (9) missing laboratory data at the corresponding time point. The specific procedure is illustrated in Figure 1. The final study cohort included 231 adult patients diagnosed with sepsis. This study was conducted in accordance with the ethical principles outlined in the Declaration of Helsinki by the World Medical Association. Ethical approval was obtained from the Ethics Committee of Nanjing Drum Tower Hospital, affiliated with Nanjing University Medical School (approval number: AF/SC‐08/03.0). Given the retrospective study design, informed consent was not required from participants.

### 2.2 Classification of Subjects and Definition

According to the Third International Consensus Definitions for Sepsis (Sepsis‐3), sepsis is a life‐threatening organ dysfunction caused by a dysregulated host response to acute infection [3]. In this study, sepsis was diagnosed based on the Sepsis‐3.0 guidelines, which define sepsis as the presence of a confirmed infection accompanied by a Sequential Organ Failure Assessment (SOFA) score of ≥ 2 points. Septic shock was defined as a subset of sepsis characterized by persistent hypotension requiring vasopressor therapy to maintain a mean arterial pressure (MAP) ≥ 65 mmHg and a serum lactate level > 2 mmol/L (18 mg/dL) despite adequate fluid resuscitation [3]. SILI was diagnosed according to Surviving Sepsis Campaign guidelines, requiring both total bilirubin (TBIL) > 34.2 μmol/L and an international normalized ratio (INR) > 1.5 [11]. Acute respiratory failure (ARF) was defined as a PaO_2_/FiO_2_ ratio < 300 mmHg within 48 h of hospital admission, accompanied by the requirement for mechanical ventilation [12]. Acute gastrointestinal injury (AGI) was characterized by impaired gastrointestinal function in critically ill patients, typically resulting from acute disease or systemic insults [13]. Acute kidney injury (AKI) was defined as a sudden decline in renal function over a short period (hours to days), characterized by an increase in serum creatinine (Cr) ≥ 0.3 mg/dL (≥ 26.5 µmol/L) within 48 h, an increase to ≥ 1.5 times the baseline within 7 days, or urine output < 0.5 mL/kg/h for ≥ 6 h [14].

### 2.3 Data Collection

Patient demographic and clinical data were collected, including name, sex, age, history of chronic diseases (hypertension, diabetes, coronary heart disease, chronic obstructive pulmonary disease, chronic kidney disease), complications (septic shock, ARF, AGI, and AKI), type of bacteria pathogen identified, infection site (blood, lungs, thoracic cavity, abdominal cavity, urinary tract, skin and soft tissue, and central nervous system), and disease severity. The highest value of laboratory indices within 24 h of admission was used to calculate the Acute Physiology and Chronic Health Evaluation II (APACHE II) score, and the same values were used to calculate the Sequential Organ Failure Assessment (SOFA) score. Laboratory parameters collected within 24 h after ICU admission included routine blood tests, human leukocyte antigen–DR (HLA‐DR), alanine aminotransferase (ALT), aspartate aminotransferase (AST), lactate dehydrogenase (LDH), TBIL, direct bilirubin (DBIL), cholinesterase (CHE), glucose, total cholesterol, triglyceride (TG), high‐density lipoprotein, low‐density lipoprotein (LDL), total protein (TP), albumin (ALB), globulin (GLB), total bile acids (TBA), Cr, uric acid (UA), INR, thrombin time (TT), d‐Dimer (D‐D), c‐reactive protein (CRP), and procalcitonin (PCT). The formulas for the combination scores of the SII, neutrophil‐to‐lymphocyte ratio (NLR), monocyte‐to‐lymphocyte ratio (MLR), and platelet‐to‐lymphocyte ratio (PLR) are as follows:

SII = Platelet Count (×10^9^/L) × Neutrophil Count (×10^9^/L)/Lymphocyte Count (×10^9^/L).

NLR = Neutrophil Count (×10^9^/L)/Lymphocyte Count (×10^9^/L)

MLR = Monocyte Count (×10^9^/L)/Lymphocyte Count (×10^9^/L)

PLR = Platelet Count (×10^9^/L)/Lymphocyte Count (×10^9^/L).

### 2.4 Statistical Analysis

Statistical analyzes were performed using IBM SPSS Statistics (version 27.0), GraphPad Prism (version 10.0), Free Statistics software (version 1.8), and R software (version 4.4.2). Normality was assessed using the Shapiro–Wilk test, and homogeneity of variance was evaluated using Levene's test. Continuous variables were expressed as median (IQR) or mean ± SD depending on their distribution, and were compared using the independent samples t‐test or Mann–Whitney *U* test as appropriate. Categorical variables were presented as frequencies and percentages and compared using the χ^2^ test. For multivariable logistic regression, variables were selected based on univariate analysis (*p* < 0.10) and collinearity diagnostics (variance inflation factor > 5 as exclusion criterion), combined with clinical relevance. Key assumptions were verified, including multicollinearity (all VIF < 3), linearity (Box–Tidwell test, all *p* > 0.05), and sample size (≥ 10 events per variable). No a priori sample size calculation was performed because this was a retrospective exploratory study using a convenience sample of available ICU patients. To evaluate the reliability of the primary effect estimate (log_2_ SII), we conducted a post‐hoc precision analysis by calculating the width of its 95% confidence interval and the upper‐to‐lower ratio. In addition, the events per variable (EPV) ratio was assessed to examine model stability; an EPV ≥ 10 is generally recommended for logistic regression. Smoothing curve analysis was performed using restricted cubic splines to assess potential non‐linearity. Subgroup analyzes were conducted using multivariable logistic regression models stratified by prespecified factors, and effect modification was evaluated using interaction terms and the likelihood ratio test. The predictive performance of SII for SILI was examined using receiver operating characteristic (ROC) curve analysis, with the area under the curve (AUC) and 95% CI calculated. All statistical tests were two‐tailed, and a *p*‐value < 0.05 was considered statistically significant.

## 3 Results

### 3.1 Clinical Characteristics at ICU Admission

Eligibility screening identified 231 patients with sepsis who fulfilled all the enrollment criteria. Based on the diagnostic criteria for SILI, the cohort was stratified into two groups: SILI (*n* = 53) and non‐SILI (*n* = 178). Baseline demographic and clinical variables are presented in Table 1. No statistically significant differences were observed between the two groups in demographic characteristics, including age and sex, APACHE II scores, comorbid conditions, or the distribution of infection types, such as those attributable to gram‐positive bacteria, viruses, fungi, bloodstream infections, skin and soft tissue infections, and urinary tract infections. Furthermore, the duration of the ICU stays also did not differ significantly between the two groups. The median age of patients with SILI was 65 years (53, 73), compared with 53 years (66, 75) in the non‐SILI group. The proportion of male patients was higher in both groups, with no statistically significant difference in sex distribution between the groups (*p* = 0.785). However, the incidence of pulmonary infections demonstrated a significant difference between the non‐SILI and SILI groups (77% vs. 52.8%, respectively; *p* < 0.001). Moreover, the SILI group exhibited a significantly higher incidence of ARF, septic shock, AGI, and AKI upon ICU admission compared to the non‐SILI group (*p* < 0.001). In addition, a statistically significant disparity in in‐hospital mortality rates was observed, with the SILI group exhibiting a mortality rate of 52.8%, which was considerably higher than the non‐SILI group's rate of 25.3% (*p* < 0.001).

**Table 1:** General characteristics between sepsis patients with liver injury and those without liver injury.

Variables	Total (*n* = 231)	Non‐SILI (*n* = 178)	SILI (*n* = 53)	*p*‐value
**Demography**				
Age (years)	66 (53,75)	53 (66,75)	65 (53,73)	0.525
Male, *n* (%)	171 (74%)	131 (73.6%)	40 (75.5%)	0.785
**Score system**				
SOFA	7 (5,11)	7 (5,10)	11 (7,13)	< 0.001
APACHE II	20 (17,25)	20 (17,25)	21 (16.5,25.5)	0.369
**Comorbidity, *n* (%)**				
Hypertension	105 (45.5)	82 (46.1)	23 (43.4)	0.732.
CHD	30 (13)	25 (14)	5 (9.4)	0.381
Diabetes	63 (27.3)	54 (30.3)	9 (17.0)	0.055
COPD	47 (20.3)	40 (22.5)	7 (13.2)	0.141
CKD	42 (18.2)	31 (17.4)	11 (20.8)	0.58
**Complication, *n* (%)**				
ARF	68 (29.4)	45 (25.3)	23 (43.4)	0.011
Septic shock	71 (30.7)	43 (24.2)	28 (52.8)	< 0.001
AGI	63 (27.3)	37 (20.8)	26 (49.1)	< 0.001
AKI	70 (30.3)	44 (24.7)	26 (49.1)	< 0.001
**Etiological infection, *n* (%)**				
Gram‐positive	67 (29.0)	48 (27.0)	19 (35.8)	0.211
Gram‐negative	162 (70.1)	132 (74.2)	30 (56.6)	0.014
Virus	33 (14.3)	29 (16.3)	4 (7.5)	0.110
Fungus	95 (41.1)	70 (39.3)	25 (47.2)	0.308
Others	4 (1.7)	3 (1.7)	1 (1.9)	0.092
**Infection site, *n* (%)**				
Blood	109 (47.2)	79 (44.4)	30 (56.6)	0.118
Lung	165 (71.4)	137 (77)	28 (52.8)	< 0.001
Thoracic cavity	3 (1.3)	2 (1.1)	1 (1.9)	0.667
Abdominal cavity	18 (7.8)	11 (6.2)	7 (13.2)	0.094
Urinary tract	28 (12.1)	21 (11.8)	7 (13.2)	0.783
Skin and soft tissue	15 (6.5)	11 (6.2)	4 (7.5)	0.723
Central nervous system	6 (2.6)	5 (2.8)	1 (1.9)	0.711
**Outcomes**				
Length of ICU stay Median (IQR), d	22 (12,38)	21 (12,35)	27 (11,43)	0.663
ICU mortality, *n* (%)	73 (31.6)	45 (25.3)	28 (52.8)	< 0.001

*Note*: Continuous variables are presented as median (IQR) and were compared using the Mann–Whitney *U* test; categorical variables are presented as n (%) and were compared using the chi‐square test or Fisher's exact test, as appropriate.

Abbreviations: AGI, acute gastrointestinal injury; AKI, acute kidney injury; APACHE II, acute physiology and chronic health evaluation II; ARF, acute renal failure; CHD, coronary heart disease; CKD, chronic kidney disease; COPD, chronic obstructive pulmonary disease; ICU, intensive care unit; IQR, interquartile range; SOFA, sequential organ failure assessment.

### 3.2 Analysis of Laboratory Data During the Initial 24 h ICU Stay

Comparative analyzes between the SILI and non‐SILI groups revealed that the SILI group exhibited significantly high levels of PCT, neutrophils, AST, LDH, TBIL, DBIL, TBA, Cr, UA, TT, INR, and D‐D (all *p* < 0.05). Conversely, the SILI group demonstrated significantly lower levels of red blood cell count, hemoglobin, lymphocytes, CHE, fasting blood glucose, TG, LDL, TP, and GLB levels. Furthermore, inflammatory markers, including SII, NLR, MLR, and PLR, were significantly higher in the SILI group than in the non‐SILI group (*p* < 0.001).

### 3.3 Multivariate Logistic Regression Analysis to Identify Risk Factors for SILI

To identify independent risk factors for SILI, multivariable logistic regression analysis was performed with candidate variables selected via a stepwise approach. Clinically relevant parameters with well‐established associations with SILI in prior literature were considered, encompassing demographic characteristics, disease severity scores, comorbidities, infection sites and laboratory parameters (Table 2). Univariate logistic regression analyzes were conducted for each candidate variable, with only those yielding a *p*‐value < 0.10 retained for further analysis. Multicollinearity among the retained variables was subsequently assessed using the variance inflation factor (VIF), and variables with a VIF > 5, indicative of severe collinearity, were excluded from the final model. After variable selection, SII, SOFA, TP, TBA, and d‐dimer were incorporated into the multivariable logistic regression model. The results identified log_2_ SII (OR 2.851, 95% CI 2.074–3.919, *p* < 0.001), SOFA (OR 1.132, 95% CI 1.020–1.257, *p* = 0.020), TP (OR 0.913, 95% CI 0.868–0.960, *p* < 0.001), and d‐dimer (OR 1.022, 95% CI 1.002–1.004, *p* = 0.035) as independent predictors of SILI, while TBA showed a borderline association (OR 1.017, 95% CI 0.997–1.038, *p* = 0.098). Notably, a higher SII was associated with an increased probability of developing SILI. Precision analysis for the primary predictor, log_2_ SII, showed a 95% confidence interval width of 1.845 (from 2.074 to 3.919) and an upper/lower ratio of 1.89, indicating acceptable precision for the odds ratio estimate. The events per variable ratio for the final model was 10.6, meeting the recommended threshold of at least 10.

**Table 2:** Laboratory test results upon ICU admission.

Variables	Total (*n* = 231)	Non‐SILI (*n* = 178)	SILI (*n* = 53)	*p*‐value
WBC, median (IQR),10^9^/L	11.1 (7.4, 15.9)	11.1 (7.3, 15.8)	10.4 (8.3, 17.2)	0.628
RBC, median (IQR), 10^12^/L	3.20 (2.71, 3.81)	3.30 (2.78, 3.94)	3.03 (2.50, 3.48)	0.027
HB, median (IQR), g/L	95 (81, 117)	97.00 (82.00, 118.25)	89.0 (74.0, 101.5)	0.005
PLT, median (IQR), 10^9^/L	127 (80, 171)	129.50 (81.50, 175.25)	109.0 (67.5, 156.0)	0.132
NE, median (IQR), 10^9^/L	8.0 (5.5, 12.3)	6.60 (4.78, 10.00)	15.50 (8.80, 22.45)	< 0.001
LYM, median (IQR), 10^9^/L	0.8 (0.4, 1.2)	0.9 (0.6, 1.4)	0.4 (0.3, 0.7)	< 0.001
MONO, median (IQR), 10^9^/L	0.5 (0.3,0.8)	0.5 (0.3, 0.8)	0.50 (0.25, 0.85)	< 0.001
ALT, median (IQR), U/L	25.3 (15,6.6)	24.35 (15.45,51.63)	30.90 (13.55, 103.35)	0.182
AST, median (IQR), U/L	38.7 (20.9, 74.0)	34.7 (19.7, 60.0)	47.5 (27.7, 180.5)	0.001
LDH, median (IQR), U/L	390 (268, 559)	375.00 (250.25, 513.25)	500 (295, 1010)	0.002
TBIL, median (IQR), μmol/L	15.5 (9.0, 25.8)	14.05 (8.08, 21.73)	32.50 (13.55, 53.85)	< 0.001
DBIL, median (IQR), μmol/L	6.1 (2.9, 12.9)	5.5 (2.7, 9.5)	13.90 (5.75, 34.30)	< 0.001
CHE, median (IQR), KU/L	3.7 (2.7, 4.7)	3.8 (2.7, 5.0)	3.2 (2.7, 4.4)	0.036
GLU, median (IQR), mmol/L	7.56 (5.69, 10.01)	7.79 (5.92, 10.50)	6.65 (5.15, 8.52)	0.012
TC, median (IQR), mmol/L	1.55 (0.99, 2.22)	1.53 (1.01, 2.12)	1.52 (0.92, 2.45)	0.822
TG, median (IQR), mmol/L	3.05 (2.30, 3.73)	3.13 (2.57, 3.80)	2.44 (2.04, 3.47)	0.001
HDL, median (IQR), mmol/L	0.57 (0.31, 0.81)	0.59 (0.36, 0.82)	0.53 (0.22, 0.78)	0.063
LDL, median (IQR), mmol/L	1.36 (0.83, 1.89)	1.51 (0.92, 1.95)	1.10 (0.61, 1.45)	< 0.001
TP, median (IQR), g/L	54.1 (48.4, 60.2)	54.70 (49.22, 60.82)	51.55 (45.68, 56.60)	0.006
ALB, median (IQR), g/L	31.65 (28.70, 35.23)	31.85 (28.88, 35.32)	31.05 (28.40, 33.50)	0.148
GLB, median (IQR), g/L	22.1 (18.0, 26.3)	22.60 (18.88, 27.20)	18.60 (16.38, 24.10)	0.004
TBA, median (IQR), μmol/L	3.6 (2.0, 8.6)	3.5 (1.9, 7.4)	6.20 (2.25, 17.15)	0.006
Cr, median (IQR), μmol/L	82 (57, 158)	73.0 (53.5, 134.0)	121.0 (75.5, 208.0)	< 0.001
UA, median (IQR), μmol/L	267 (177, 388)	251.0 (172.8,352.0)	338.0 (197.0, 430.5)	0.016
INR, median (IQR)	1.21 (1.09, 1.45)	1.17 (1.08, 1.36)	1.48 (1.17, 1.83)	< 0.001
TT, median (IQR), s	16.9 (15.8, 19.3)	16.6 (15.7, 18.4)	18.4 (16.8, 28.0)	< 0.001
APTT, median (IQR), s	31.40 (27.43, 40.88)	30.50 (21.70, 36.10)	38.30 (30.80, 52.75)	< 0.001
D‐D, median (IQR)	6.61 (2.88, 13.71)	6.23 (2.86, 11.41)	8.76 (3.54, 26.73)	0.019
CRP, median (IQR), mg/L	83.30 (40.40, 145.70)	94.60 (41.85, 146.25)	92.75 (50.38, 194.88)	0.485
PCT, median (IQR), ng/ml	1.06 (0.27, 5.60)	0.98 (0.21, 5.59)	5.42 (1.05, 18.41)	< 0.001
HLA‐DR, median (IQR), %	33.3 (19.2, 52.9)	32.00 (19.70, 46.35)	22.60 (15.15, 51.15)	0.293
SII, median (IQR)	1103.38 (570.83,2593.75)	945.23 (514.10, 1717.24)	5005.50 (2608.19, 7708.80)	< 0.001
NLR, median (IQR)	8.55 (5.33, 20.57)	7.36 (4.92, 12.63)	43.25(26.76, 65.13)	< 0.001
MLR, median (IQR)	0.57 (0.33, 1.00)	0.50 (0.29, 0.81)	1.07 (0.59, 1.81)	< 0.001
PLR, median (IQR)	161.00 (93.75, 280.00)	130.63 (82.40, 226.58)	280 (184.15, 415.00)	< 0.001

*Note*: Continuous variables are presented as median (IQR) and were compared using the Mann–Whitney *U* test due to non‐normal distribution; categorical variables were compared using the chi‐square test.

Abbreviations: ALB, albumin; ALT, alanine aminotransferase; AST, aspartate aminotransferase; CHE, cholinesterase; Cr, creatinine; CRP, C‐reactive protein; D‐D, d‐dimer; DBIL, direct bilirubin; GLB, globulin; GLU, glucose; Hb, hemoglobin; HDL, high‐density lipoprotein; HLA‐DR, human leukocyte antigen–DR; INR, international normalized ratio; LDH, lactate dehydrogenase; LDL, low‐density lipoprotein; LYM, lymphocyte; MLR, monocyte‐to‐lymphocyte ratio; MONO, monocyte; NE, neutrophil; NLR, neutrophil‐to‐lymphocyte ratio; PCT, procalcitonin; PLR, platelet‐to‐lymphocyte ratio; PLT, platelet; RBC, red blood cell; SII, systemic immune‐inflammation index; TBA, total bile acids; TBIL, total bilirubin; TC, total cholesterol; TG, triglyceride; TP, total protein; TT, thrombin time; UA, uric acid; WBC, white blood cell.

### 3.4 Curve Fitting and Subgroup Analyzes to Determine the Associations Between SII and SILI

**Figure 2:**
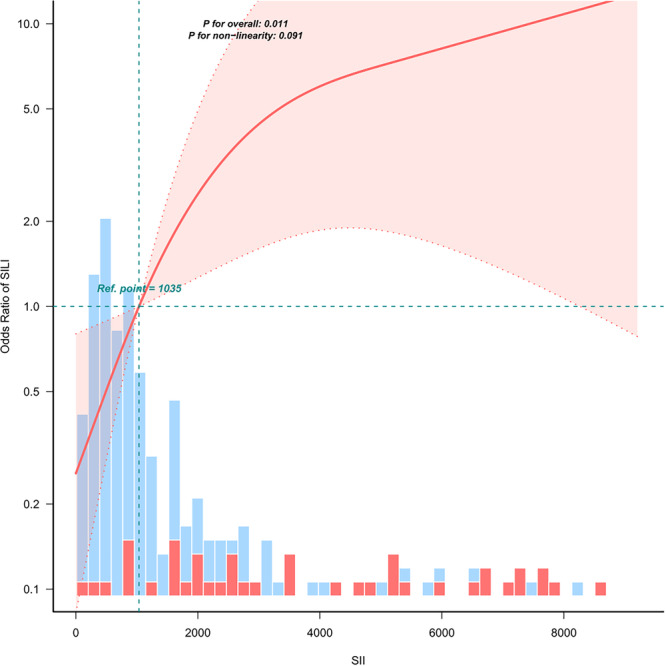
Association between initial SII level and SILI in adult patients with sepsis. SII systemic immune inflammatory index; SILI Sepsis‐induced liver injury, the red solid curve represents the odds ratio, and the coral area indicates its 95% confidence interval; the blue histograms show the percentage of non‐SILI participants belonging to each level of initial SII value; the red histograms show the percentage of SILI participants belonging to each level of initial SII value.

**Figure 3:**
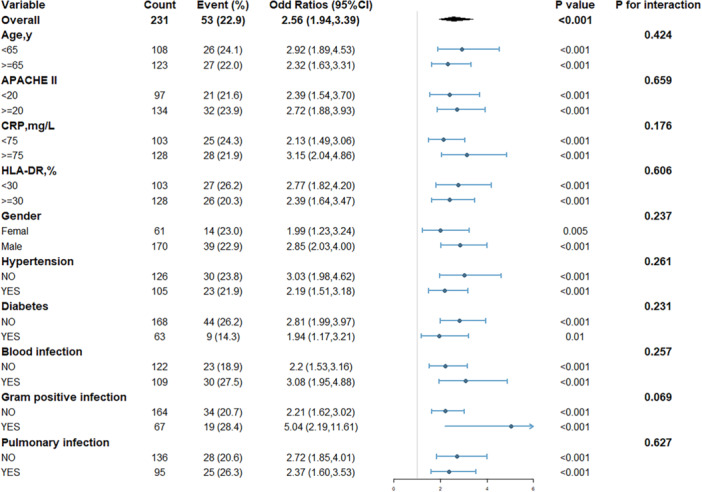
Subgroup analysis of the association between SII and SILI risk. APACHE II, acute physiology and chronic health evaluation II; CRP, C‐reactive protein; HLA‐DR, human leukocyte antigen‐DR.

After adjustment according for potential confounding variables, smoothing curve analysis revealed a linear association between the initial SII and SILI (p for non‐linearity = 0.091; Figure 2). Given the heterogeneity of the study population, participants were stratified based on age (> 65 years, ≤ 65 years), APACHE II score (< 20, ≥ 20), CRP levels (< 75 mg/L, ≥ 75 mg/L), HLA‐DR levels (< 30%, ≥ 30%), sex, comorbidities, and infection status. As shown in Figure 3, the SII value remained significantly associated with sepsis‐induced liver dysfunction across all subgroups (*p* < 0.05). Further interaction analysis revealed no significant multiplicative interactions for age (*p* = 0.424), APACHE II (*p* = 0.659), CRP (*p* = 0.176), HLA‐DR (*p* = 0.606), sex (*p* = 0.237), hypertension (*p* = 0.261), diabetes (*p* = 0.231), bloodstream infection (*p* = 0.257), gram positive infection (*p* = 0.069), and pulmonary infection (*p* = 0.627). Table 3.

**Table 3:** Multivariate logistic regression analysis of risk factors for sepsis‐induced liver injury.

Variables	OR	95% CI for OR	*p*‐value
Log_2_SII	2.851	(2.074, 3.919)	< 0.001
SOFA	1.132	(1.020, 1.257)	0.020
TP	0.913	(0.868, 0.960)	< 0.001
TBA	1.017	(0.997, 1.038)	0.098
D‐D	1.022	(1.002, 1.004)	0.035

*Note*: Multivariable logistic regression analysis was performed with variable selection based on univariable *p* < 0.10, clinical relevance, and collinearity diagnostics (VIF < 5). Model fit assessed by Hosmer–Leme show test.

Abbreviation: CI, confidence interval; D‐D, d‐dimer; OR, odds ratio; SII, systemic immune‐inflammation index; SOFA, sequential organ failure assessment; TBA, total bile acids; TP, total protein.

### 3.5 Predictive Performance of SII for SILI

**Figure 4:**
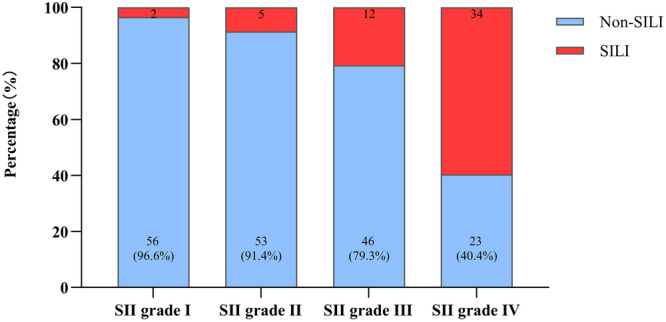
The incidence rates of sepsis‐induced liver injury in different SII‐based stratification groups. SII, systemic immune‐inflammation index; SII grade I (≤ 570.83), SII grade II (572.25–1103.38), SII grade III (1110–2593.75), SII grade IV (≥ 2613).

**Figure 5:**
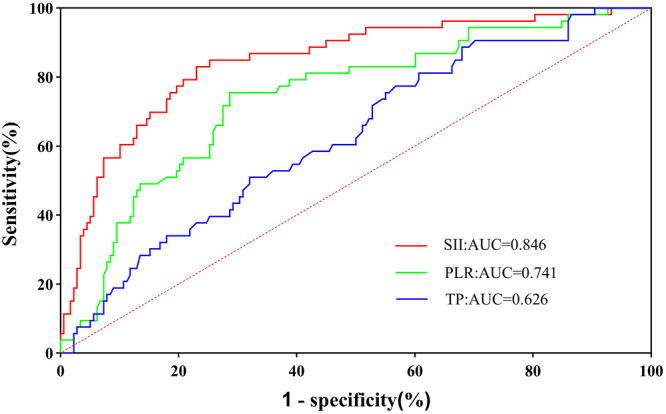
ROC curves of SII, PLR, TP and urea for predicting the development of SILI. AUC, area under the curve; PLR, platelet‐to‐lymphocyte ratio; SII, systemic immune‐inflammation index; TP, total protein.

For descriptive purposes only, patients were stratified into four groups according to their SII quartile ranges. Figure 4 illustrates the relationship between the SII grades and SILI incidence, with the latter increasing progressively with higher SII grade (Grade I, 3.4%; Grade II, 8.6%; Grade III, 20.7%; Grade IV, 59.6%). Notably, in the primary multivariable logistic regression analysis, SII was analyzed as a continuous variable (log_2_‐transformed) without categorization, and smoothing curve analysis confirmed no significant non‐linearity (P for non‐linearity = 0.091, Figure 2). Furthermore, ROC curve analyzes for SII, PLR, TP, and urea were performed with results indicating that the P‐values for the other three indicators were all < 0.05, except for urea (*p* = 0.149). Table 4 presents the sensitivity, specificity, AUC, and optimal cutoff points. In terms of sensitivity, SII (83%) was slightly lower than TP (88.7%), while in specificity, SII (77%) outperformed the other indicators. The AUC values were 0.846, 0.741, and 0.626 for SII, PLR, and TP, respectively, with Figure 5 showing that SII had the largest AUC among all indicators. Although these indicators demonstrated good predictive performance for the SILI, the SII clearly exhibited a superior predictive value over PLR and TP.

**Table 4:** Cutoff values and AUC for each parameter for predicting SILI.

Variable	AUC (95%CI)	Sensitivity (%)	Specificity (%)	Cut‐off	*p*‐value
SII	0.846 (0.784–0.907)	83.00	77.00	1712	< 0.001
PLR	0.741 (0.666–0.817)	75.50	71.30	192.36	< 0.001
TP	0.626 (0.543–0.708)	88.7	32.00	59.55	0.006
Urea	0.565 (0.478–0.653)	0.68	0.51	0.19	0.149

*Note*: ROC curve analysis was performed to evaluate predictive performance. Optimal cut‐off values were determined by maximizing the Youden index.

Abbreviations: AUC, area under the receiver operating characteristic curve; CI, confidence interval; SII, systemic immune‐inflammation index; PLR, platelet‐to‐lymphocyte ratio; TP, total protein.

## 4 Discussion

This study systematically investigated the association between the inflammatory immune index SII and the risk of SILI in adult patients with sepsis using routinely available clinical data and statistical analyzes. The findings provide objective evidence supporting the early risk stratification of SILI and establish a potential theoretical foundation for developing individualized treatment strategies for sepsis management.

Our analysis revealed a SILI incidence of 23% and a 52.8% in‐hospital mortality rate. Although the SILI incidence in our study differed from that reported by Cui et al. [15], the mortality rates were similar. This discrepancy may be attributed to the exclusion criteria used by Cui et al., which included patients who died or were discharged within 24 h of ICU admission. Previous studies have highlighted the prognostic value of the SOFA score in patients with sepsis and its ability to predict SILI. Wang et al. reported significantly higher SOFA scores in the SILI group (11 [7, 8, 9, 10, 11, 12, 13] vs. 5 [4, 5], *p* = 0.005) [16], consistent with our findings and a clinical study of children with sepsis under 19 years of age [17], which demonstrated that higher SOFA scores correlated positively with greater SILI incidence and sepsis severity. Multivariate logistic regression analysis revealed that SII, PLR, and TP were significantly associated with SILI occurrence. The ROC curve further substantiated the reliability of early SII as a predictive tool for the potential occurrence of SILI in patients with sepsis.

Sepsis represents a dysregulated host response to invading pathogens, characterized by a hyperinflammatory phase in the early stages. Common clinical manifestations include fever, tachycardia, tachypnea, and leukocytosis, collectively referred to as systemic inflammatory response syndrome [18]. The liver is the largest metabolic organ in the human body and a critical component of the immune system. However, excessive inflammation and immune dysregulation can lead to hepatic damage, a condition known as SILI. The pathological manifestations of SILI include steatosis, cholangitis, intrahepatic cholestasis, periportal inflammation, and hepatocyte apoptosis [19, 20, 21]. During infection, pathogen‐associated molecular patterns, such as lipopolysaccharide and bacterial DNA, activate macrophages via the TLR4/NF‐κB signaling pathway, triggering the release of high mobility group box 1 (HMGB1) [6]. In turn, HMGB1 binds to its cognate receptors and facilitates cytosolic LPS translocation, thereby inducing apoptosis in hepatocytes. Moreover, HMGB1‐mediated pyroptosis of hepatic Kupffer cells exacerbates acute liver injury during sepsis. In addition, gram‐positive bacterial infections have been shown to significantly increase the risk of acute liver injury [20], primarily by hyperactivating both immune and non‐immune cells, triggering a cytokine storm during sepsis. This results in circulating cytokines and other inflammatory mediators directly promoting hepatocyte apoptosis and necrosis.

To facilitate early detection and prevention of sepsis progression, clinicians routinely obtain blood samples and perform laboratory testing to evaluate infection severity. Currently, commonly inflammatory indicators include white blood cell count (WBC), CRP, and PCT [22, 23, 24]. These markers can assist clinicians in rapidly and conveniently assessing the inflammatory and immune status of patients. Although useful, these indicators are often subject to intrinsic or extrinsic interference or may exhibit delayed responses. Sander et al. reported that while WBC count and its differentials can dynamically monitor infection trends and preliminarily determine infection type, they may not be significantly increased in the early stages of infection, leading to delayed diagnosis [22]. Similarly, CRP, as an acute‐phase reactant, begins to rise 6–12 h after infection or inflammation onset and peaks at 24–48 h, resulting in low sensitivity for early infections [23]. Additionally, CRP levels did not correlate linearly with the infection severity, as both mild and severe cases can exhibit similar CRP elevations [24]. Owing to the limitations of these indicators as stand‐alone diagnostic criteria for inflammation, the combined detection of biomarkers such as platelets, PCT, neutrophils, and ALB has gained increasing attention for evaluating various diseases [25, 26, 27].

In 2014, Hu et al. formulated a composite SII biomarker, derived from three routine hematologic parameters (lymphocytes, neutrophils, and platelets), as a quantitative measure of systemic inflammation [8]. The SII demonstrated a significantly superior predictive ability for recurrence in patients undergoing radical resection of hepatocellular carcinoma compared to NLR, PLR, and other conventional parameters. Neutrophils, the predominant phagocytic mediators of innate immunity, undergo prioritized recruitment to infection sites, where they initiate the earliest antimicrobial effector mechanisms and eliminate pathogens through phagocytosis and the release of neutrophil extracellular traps [28]. In sepsis, however, neutrophils exhibit delayed apoptosis coupled with compromised chemotactic capacity, leading to intravascular sequestration owing to dysfunctional transendothelial trafficking detected via serological assays [29]. Concurrently, T‐lymphocyte dysfunction has been established as a critical pathophysiological hallmark of sepsis‐associated immunosuppression. Accumulating evidence suggests that apoptotic pathways play a pivotal role in sepsis‐induced immunosuppressive mechanisms [30], characterized by marked depletion of lymphocyte populations in both the peripheral circulation and lymphoid organs, and a strong positive correlation between the apoptotic index and disease severity [31]. The precise mechanistic role of platelets in sepsis pathogenesis remains unclear, though growing evidence indicates that sepsis induces platelet hyperactivation [32]. Notably, Cao et al. demonstrated that elevated platelet aggregation within 24 h of hospital admission serve as an independent predictor of 28‐day mortality in patients with sepsis [33]. Similarly, a prospective cohort study involving over 100,000 individuals from the general population found that the risk of psoriasis diagnosis during follow‐up increased with higher SII values. Furthermore, the combined assessment of the SII, NLR, and CRP levels revealed that low‐grade systemic inflammation is an independent risk factor for psoriasis [27]. The SII quantifies the inflammatory burden and reflects the balance between the innate and adaptive immune systems. Its inclusion in the present study in the SILI analysis supplements early predictive indicators for SILI.

This study had several methodological limitations. First, retrospective data collection from a single medical center yielded a relatively small study population, which may have introduced bias owing to regional variations, a limited temporal scope, and resource constraints. Second, the study population consisted solely of ICU patients, whose physiological parameters may exhibit a narrower range of fluctuations than those of general ward patients, potentially limiting generalizability to less critically ill patients. Third, only baseline SII values of patients with sepsis at ICU admission were analyzed, without further investigation of dynamic changes in SII levels over time. Finally, the prognostic value of the relationship between the SII and SILI remains unclear. Therefore, large‐scale prospective studies and longitudinal evaluations are needed in the future to validate the diagnostic value of the SII for SILI.

In conclusion, this study demonstrated a robust association between SII and SILI in adult patients with sepsis, with SII emerging as the strongest predictive indicator among the indices examined. To the best of our knowledge, this study is the first to validate the use of SII as a predictive biomarker for SILI. This validation offers a window for preventive measures that could substantially improve sepsis outcomes.

## Author Contributions

**Zimeng Qin:** conceptualization, methodology, software, data curation, investigation, validation, formal analysis, writing – original draft. **Jiaqi Li:** methodology, software, investigation, formal analysis. **Yijiang Liu:** validation, formal analysis, software, data curation. **Beiyuan Zhang:** writing – review and editing, funding acquisition, conceptualization, supervision, visualization, project administration. **Wenkui Yu:** writing – review and editing, funding acquisition, conceptualization, supervision, visualization, project administration.

## Ethics Statement

The studies involving human participants were reviewed and approved by the Medical Ethics Committee of Nanjing Drum Tower Hospital (approval number: AF/SC‐08/03.0). The Institutional Review Board (IRB) waived informed consent requirements, all data were completely anonymized and the study presented minimal risk. All data were used exclusively for research purposes with strict confidentiality safeguards.

## Conflicts of Interest

The authors declare no conflicts of interest.

## Data Availability

The data that support the findings of this study are available from the corresponding author upon reasonable request.
